# Specific and Aspecific Molecular Checkpoints as Potential Targets for Dismantling Tumor Hierarchy and Preventing Relapse and Metastasis Through Shielded Cytolytic Treatments

**DOI:** 10.3389/fcell.2021.665321

**Published:** 2021-07-06

**Authors:** Giovanni Manzo

**Affiliations:** Chemical-Biological Department of IISS, Maglie, Italy

**Keywords:** cancer stem cell, molecular checkpoints, extracellular vesicles, embryo, therapy

## Abstract

I have recently theorized that several similarities exist between the tumor process and embryo development. Starting from an initial cancer stem cell (CSC_0_), similar to an embryonic stem cell (ESC), after implantation in a niche, primary self-renewing CSCs (CSC_1_s) would arise, which then generate secondary proliferating CSCs (CSC_2_s). From these epithelial CSCs, tertiary mesenchymal CSCs (CSC_3_s) would arise, which, under favorable stereotrophic conditions, by asymmetric proliferation, would generate cancer progenitor cells (CPCs) and then cancer differentiated cells (CDCs), thus giving a defined cell heterogeneity and hierarchy. CSC_1_s–CSC_2_s–CSC_3_s–CPCs–CDCs would constitute a defined “tumor growth module,” able to generate new tumor modules, forming a spherical avascular mass, similar to a tumor sphere. Further growth *in situ* of this initial tumor would require implantation in the host and vascularization through the overexpression of some aspecific checkpoint molecules, such as CD44, ID, LIF, HSP70, and HLA-G. To expand and spread in the host tissues, this vascularized tumor would then carry on a real growth strategy based on other specific checkpoint factors, such as those contained in the extracellular vesicles (EVs), namely, microRNAs, messenger RNAs, long non-coding RNAs, and integrins. These EV components would be crucial in tumor progression because they can mediate intercellular communications in the surrounding microenvironment and systemically, dictating to recipient cells a new tumor-enslaved phenotype, thus determining pre-metastatic conditions. Moreover, by their induction properties, the EV contents could also frustrate in time the effects of cytolytic tumor therapies, where EVs released by killed CSCs might enter other cancer and non-cancer cells, thus giving chemoresistance, non-CSC/CSC transition (recurrence), and metastasis. Thus, antitumor cytotoxic treatments, “shielded” from the EV-specific checkpoints by suitable adjuvant agents, simultaneously targeting the aforesaid aspecific checkpoints should be necessary for dismantling the hierarchic tumor structure, avoiding recurrence and preventing metastasis.

## Introduction

**FIGURE 1:**
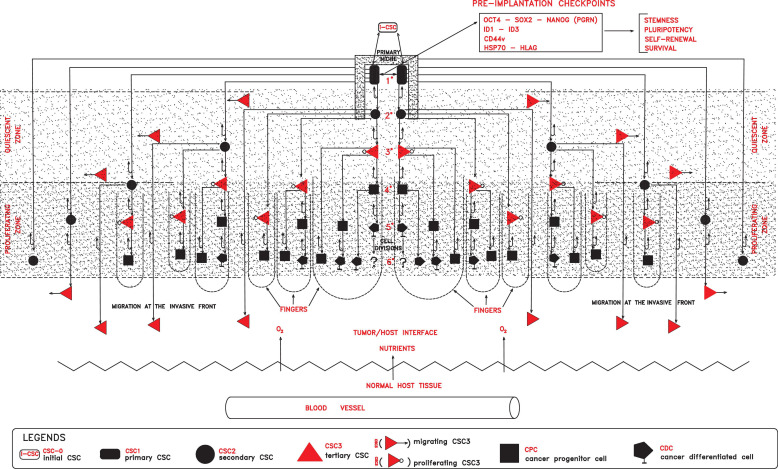
In a primary niche, primary self-renewing cancer stem cells (CSC_1_s) endowed with stemness properties, due to defined preimplantation checkpoint factors (OCT4, SOX2, NANOG, ID, CD44, HSP70, and HLA-G), would generate progressively secondary proliferating CSCs (CSC_2_s), tertiary mesenchymal CSCs (CSC_3_s), cancer progenitor (CPCs), and cancer differentiated (CDCs) cells, globally forming a tumor module where two zones would lie (quiescent and proliferating). In the proliferating zone, more external with normoxia conditions, CSC_3_s and CPCs would proliferate, generating cell cord-finger structures on the invasive front at the tumor/host interface. On the other hand, in the quiescent zone, more internal with hypoxic conditions, quiescent CSC_3_s would be induced to migrate peripherally, seeding new local niches in the normal host tissues. All these processes, finally, would result in a tumor module with a defined cell heterogeneity and hierarchy.

**FIGURE 2:**
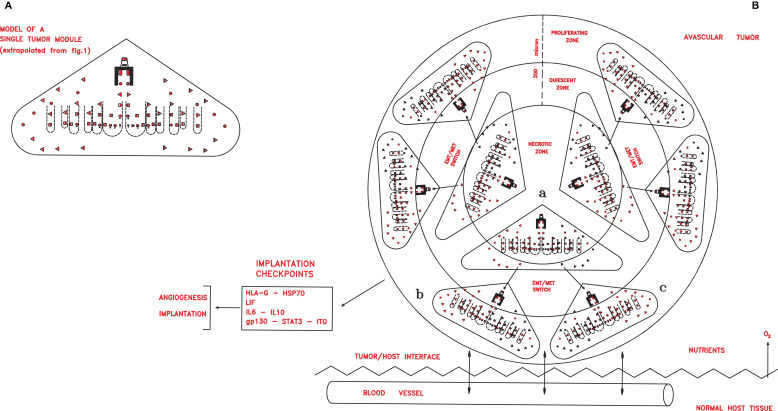
**(A)** A simplified scheme of a single tumor module, obtained from Figure 1 extrapolating only the symbols depicting the different cancer cells shown in the legends of the Figure 1. In an avascular tumor **(B)**, initial tumor modules (a), located in the central zone, would generate new tumor modules (b,c) in a spherical structure of about 400 microns. Since the nutrient diffusion limits are about 200 μm, further growth would imply cell death in the central zone, while in the peripheral zone growth could occur through neoangiogenesis and vascularization, which allow tumor implantation in the host tissues, thanks to defined checkpoint factors (HLA-G, HSP70, IL-6, and gp130–STAT3–ITG).

**FIGURE 3:**
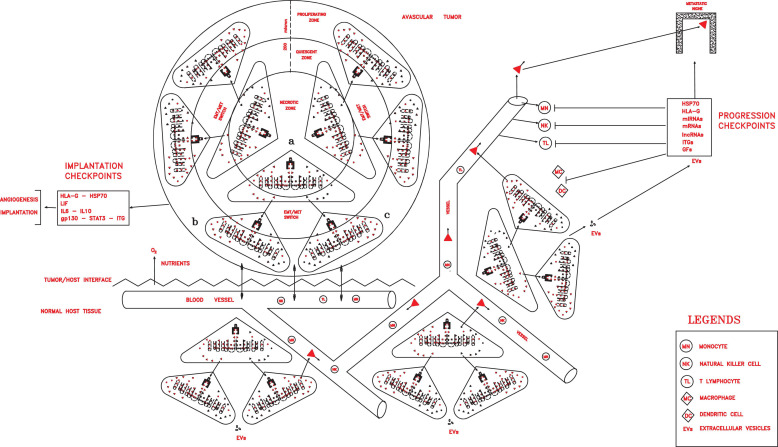
Once implanted and vascularized, a tumor could grow rapidly, invading host tissues, spreading in the circulation, and installing in distant pre-metastatic niches, thanks to progression checkpoint factors (HLA-G, HSP70, miRNAs, mRNAs, lncRNAs, ITGs, and GFs) released in extracellular vesicles (exosomes in particular) by cancer stem cells (CSCs) and cancer progenitor cells (CPCs) and able to impair all immune cells.

I have recently theorized that several similarities exist between the tumor process and embryo development (Manzo, 2019). Starting from an initial cancer stem cell (i-CSC/CSC_0_), similar to an embryonic stem cell (ESC) without genomic homeostasis (para-ESC), after implantation in a niche, primary self-renewing cancer stem cells (CSC_1_s) would arise, corresponding to epiblast cells. CSC_1_s would then generate secondary proliferating CSCs (CSC_2_s), equivalent to hypoblast cells. From CSC_1_s and CSC_2_s, with an epithelial phenotype, tertiary CSCs (CSC_3_s) with a mesenchymal phenotype would arise, corresponding to the mesodermal precursors at the primitive streak (PS). Under favorable stereotrophic conditions (normoxia), CSC_3_s would undergo asymmetric proliferation and pre-differentiation into cancer progenitor cells (CPCs) and then into cancer differentiated cells (CDCs), thus giving a defined cell heterogeneity and hierarchy (Marjanovic et al., 2013; Singh et al., 2015; Bradshaw et al., 2016), mimicking an ectopic rudimentary somito-histo-organogenesis process (Reya et al., 2001; Gibbs, 2009; Ma et al., 2010). In contrast, under unfavorable stereotrophic conditions (hypoxia), CSC_3_s would delaminate and migrate as quiescent micro-metastases, mimicking embryonic morphogenetic movements and localizing in metastatic niches (Cabrera et al., 2015; Singh et al., 2015; Yang et al., 2018). Here, specific signals, similar to those occurring in the embryonic inductions, would induce an epithelial–mesenchymal transition (EMT)/mesenchymal–epithelial transition (MET) switch (Thiery et al., 2009; Liu et al., 2014), allowing the reversion of quiescent CSC_3_s into proliferating CSC_1_s. These cells would be able to generate macro-metastases with the same cell hierarchy as their primary tumors (Marjanovic et al., 2013). Within this proliferation model, CSC_1_s–CSC_2_s–CSC_3_s–CPCs–CDCs would constitute a defined “tumor growth module” with a cord-finger structure (Manzo, 2019, 2020; Figure 1), where it is possible to find well-defined mathematical relationships between CSCs (CSC_1_s, CSC_2_s, and CSC_3_s) and non-CSCs (CPCs and CDCs) at each (*n*) cell division (Manzo, 2020). A tumor growth module would generate new modules after about 10 division cycles, when the cell number would become presumably too large for survival under unfavorable stereotrophic conditions (Hamilton and Rath, 2019; Manzo, 2020). Such a modular growth process seems to occur also when CSCs, cultured *in vitro* in the absence of implantation conditions, form solid, round cellular structures with a diameter of about 50–250 μm, named tumor spheres, displaying a modular growth behavior similar to that of avascular tumors *in vivo* (Johnson et al., 2013; Vinnitsky, 2014). Such tumor growth, occurring by reiterative production of defined cell modules, would generate an initial spherical avascular mass (Figure 2). This might expand until it reaches a diameter of approximately 400 μm since diffusion and the supply of nutrients and oxygen at the core cells are not possible beyond about 200 μm (Hamilton and Rath, 2019). Beyond this limit, avascular tumor growth could occur only with a simultaneous death of the core cells (Hamilton and Rath, 2019). Up to this point, the tumor process would be similar and equivalent to that of a preimplantation blastocyst (Manzo, 2020). Now, further tumor growth would require implantation and vascularization for the oxygen and nutrient supply by the host microenvironment, like in embryo development. In such a way, an avascular tumor might become a vascularized tumor, where, together with nutrients, immune cells also arrive (Figure 3). Therefore, vascular tumor cells need to defend themselves from immune cells for survival, like what a post-implantation semi-allogeneic blastocyst do from maternal immune cells (Yao et al., 2005; Gregori et al., 2015; Manzo, 2019). On the other end, a tumor needs to expand in the host tissues. To this end, it would carry out a real growth strategy based on defined structures, such as extracellular vesicles (EVs) with their contents (Jurj et al., 2020), able to impair the host immune system and induce tumor growth, allowing tumor progression and metastases (Vader et al., 2014; Lin and Yan, 2015, 2019; Han et al., 2019), mimicking ectopic rudimentary organ portions. Now, I intend to point out and analyze crucial factors in the different phases of the cancer process for detecting potential checkpoints to be targeted in therapeutic treatments.

## Theoretical Checkpoints in the Different Phases of the Cancer Process

In the cancer process, three stages could be distinguished: preimplantation avascular tumor, implantation vascularized tumor, and progressing metastatic tumor.

### Avascular Tumors (Preimplantation Tumors)

In this first phase, molecular checkpoints playing a major role could be factors able to confer to cells of an initial tumor persistent stemness, self-renewal, pluripotency, survival, and apoptosis inhibition, such as OCT4, NANOG, SOX2, STAT3, CD44, ID, HLA-G, and HSP70 factors (Figure 1).

#### OCT4, SOX2, and NANOG

OCT4, SOX2, and NANOG are well known to constitute a sort of molecular engine in both embryogenesis and cancer genesis, regulating the so-called pluripotency gene regulatory network (PGRN), sustaining stemness, pluripotency, self-renewal, and reprogramming (Festuccia et al., 2013).

#### CD44

This factor is a cell surface protein constituting a signal platform that regulates the expressions of genes related to proliferation, migration, survival, and invasion (Williams et al., 2013; Yan et al., 2015). The CD44 variant isoforms (CD44v3, CD44v6, CD44v8, etc.), absent in normal tissues, seem to be restricted to aggressive tumors and have crucial roles in the regulation of stemness, self-renewal, tumor initiation, metastasis, and chemoresistance (Zeilstra et al., 2014; Chanmee et al., 2015). They are expressed both in epithelial (ALDH1^+^CD44^+^Ki67^+^, hypothetical CSC_1_s) and mesenchymal (ALDH1^–^CD44^+^Ki67^–^, hypothetical CSC_3_s) CSCs (Liu et al., 2014; Manzo, 2019). CD44v isoforms are critical during EMT in cancer progression (Xu et al., 2015; Yan et al., 2015; Chen C. et al., 2018). The CD44/STAT3 complex induces both epithelial (proliferation) and mesenchymal (migration) features (Yan et al., 2015), and a transforming growth factor beta (TGF-β)-induced CD44^high^/ID1^high^ expression occurs in glioma-initiating cells (Anido et al., 2010). Moreover, a positive feedback couples Ras activation and CD44v (Cheng et al., 2006; Krishnamachary et al., 2012). Thus, CD44v could be a sort of molecular trigger of a direct reprogramming (Su et al., 2011) in the EMT/MET switch of quiescent CSC_3_s to self-renewing CSC_1_s.

#### ID1 and ID3

ID1 and ID3 are expressed only in embryonic and cancer cells, but not in most adult tissues. ID1 proteins have multiple roles in several processes, such as in the implantation of CSCs (CSC_1_s) in primary and metastatic niches, apoptosis inhibition, survival and growth, angiogenesis, and chemoresistance (Lyden et al., 1999; Ling et al., 2006; Niola et al., 2012; Nair et al., 2014). ID1 proteins, in association with ubiquitous E proteins, prevent CSCs from differentiating (Ling et al., 2006), thus determining the crucial “blocking event” that would confer to CSC_1_s persistent stemness, with self-renewal and pluripotency capacities, reiteratively feeding the tumor. ID1 and ID3 would be necessary for TIC (tumor-initiating cell) functions in the genesis of both primary tumors and metastases, sustaining proliferation *via* p21 (Gupta et al., 2007; O’Brien et al., 2012). ID1 and ID3 are required for angiogenesis and the vascularization of tumor xenografts (Lyden et al., 1999; Teo et al., 2020) necessary for macro-metastases development.

#### HSP70s and HLA-Gs

These proteins are precociously expressed in embryogenesis and cancer genesis, but only transiently during mitosis (70-kDa heat shock proteins, HSP70s) or in a few defined organs (human leukocyte antigen G, HLA-Gs) in adults (Stangl et al., 2011; Tilburgs et al., 2015). In preimplantation embryos and initial avascular tumors, they would constitute protection and survival systems, both by preventing apoptosis (HSP70) (Samali and Cotter, 1996) and defending from adverse host microenvironments (HSP70 and membrane HLA-G1), or also a system for invading and colonizing the host tissues (soluble HLA-G5) (Rouas-Freiss et al., 2007; Sheu and Shih, 2010). HLA-Gs are expressed in embryonic and tumor mesenchymal cells (CSC_3_s) and in progenitor cells (CPCs) (Yen et al., 2009). A small amount of HSP70s is necessary for preimplantation embryogenesis (Luft and Dix, 1999). HSP70s can be expressed on the cell surface or exported in the circulation (Shu and Huang, 2008). Highly metastatic tumors, but not their primary counterparts, express membrane HSP70s. HSP70+ tumors actively release exosomes with an HSP70+ surface (Stangl et al., 2011), which might act in tumor protection, survival, and spread. During embryo development, HLA-Gs orchestrate the early interaction of human trophoblasts with the maternal niche for implantation (Gregori et al., 2015); after implantation, HLA-Gs are expressed in the endothelial cells of developing vessels, mesenchymal cells, and progenitor cells (Hunt et al., 2005; Yao et al., 2005; Verloes et al., 2011). Tumor and mesenchymal cells secrete HLA-Gs in EVs (Yen et al., 2009; Burrello et al., 2016; Rebmann et al., 2016). HLA-G expression has been shown in many cancer types, both in primary and metastatic tumors, mainly as soluble HLA-G5, but also associated with EVs (Rebmann et al., 2003, 2016). HLA-G expression is induced by hypoxia *via* HIF-1a (Rebmann et al., 2003) and is upregulated by interleukin (IL)-10 with an autocatalytic feedback (Urosevic et al., 2001, 2002). HLA-G induces IL-6 production (Urosevic et al., 2002) and, thus, the activation of the gp130–JAK–STAT3 pathway, regulating proliferation, migration, invasion, and angiogenesis, namely, tumor progression and metastasis (Kamran et al., 2013). Notably, HLA-G expression is also induced and upregulated by chemotherapeutic agents (Rouas-Freiss et al., 2003; Yan et al., 2005).

### Vascular Tumors (Implanted Tumors)

In this second phase, the major molecular checkpoints might be factors able to confer to cancer cells implantation properties in primary local niches and angiogenesis for vascularization, such as ID1, ID3, leukemia inhibitory factor and its receptor (LIF/LIFr), IL-6/IL-6r, IL-10, gp130, Janus kinase (JAK), signal transducer and activator of transcription 3 (STAT3), HSP70, and HLA-G factors (Figure 2). LIF/LIFr would have an autocrine/paracrine function in embryo implantation (Cullinam et al., 1996) by activating and regulating the gp130–JAK–STAT3, AKT, and ERK1–2 MAPK signal pathways that induce the expression of integrin a5b1, realizing implantation, endothelial proliferation, and subsequent angiogenesis–vascularization (Cheng et al., 2001; Sherwin et al., 2002; Park et al., 2003). LIF–gp130–STAT3 pathway activation is also linked to IL-6 and IL-10, as well as to HLA-G that interacts with these factors (Urosevic and Dummer, 2003; Yue et al., 2015). In LIF knockout mice and in “*in vitro*” fertilized human embryos lacking HLA-G5, implantation does not occur (Fuzzi et al., 2002). In many cancer types, including melanomas, skin, kidney, prostate, and pancreatic cancers, a LIF signal is expressed at high levels, inducing an autocrine/paracrine cell proliferation, like in embryo implantation (Cullinam et al., 1996; Kellokumpu-Lehtinen et al., 1996). The LIF amount secreted by a tumor seems to regulate cancer genesis (Guo et al., 2015). In solid tumors, LIF expression is induced by hypoxia *via* HIF-2a and by TGF-β (Yue et al., 2015). A high LIFr expression is crucial for tumor implantation: analysis of 90 nevi and 441 melanomas shows that LIFr expression is low for all nevus stages, starts to increase in dysplastic nevi, becomes higher in implanted melanomas, and is highest in metastatic melanomas (Guo et al., 2015). Thus, LIF/LIFr, together with interacting IL-6/IL-6r, IL-10, and HLA-G, would be crucial for implantation, autocrine/paracrine growth, the vascularization of primary tumors *via* gp130–JAK–STAT3 pathways, as well as for genesis of mesenchymal metastasizing cells (Yan et al., 2015).

### Progressing Tumors (Metastatic Tumors)

In this tumor phase, many different molecular factors and structures could constitute crucial checkpoints of the cancer process, which are able to favor local growth and angiogenesis and confer properties of invasion and migration, protection and escape from immune surveillance, detection and invasion of specific pre-metastatic niches, and the capacity of dictating tumor conditions in the surrounding microenvironment. Such factors could include CD44v, ID1, ID3, HSP70, HLA-G, LIF/LIFr, IL-6, IL-10, and EVs with their contents, such as exosomal integrins (ITGs), microRNAs (miRNAs), messenger RNAs (mRNAs), long non-coding RNAs (lncRNAs), and growth factors (GFs) (Figure 3).

Essentially, three types of EVs are released by tumor cells, marked on the basis of their size: (a) exosomes, (b) microvesicles, and (c) apoptotic bodies. Exosomes are released constitutively and/or upon cell activation and hypoxia induction, have a size of about 40–150 nm in diameter, and have an endocellular origin from early endosomes. Exosomes enter the recipient cells by an inverse mechanism of endocytosis within endosomes, from which they release their contents in the recipient cell cytoplasm (Vader et al., 2014). Microvesicles have a size of about 50–2,000 nm in diameter, have a membrane origin, are released like the exosomes, and enter the recipient cells by direct membrane fusion, direct endocytosis, or after interaction of their ligands with specific cell surface receptors (Vader et al., 2014). Apoptotic bodies have a size of about 50–50,000 nm, are formed by random blebbing of the plasma membrane, are released by apoptotic tumor cells, and may contain nuclear fragments with DNA and histones, as well as fragments of cytoplasmic organelles, which they can transfer also to normal cells, leading to the development of a full tumorigenic potential (Vader et al., 2014). EVs contain nucleic acids (DNA fragments, oncogenes, mRNAs, miRNAs, and lncRNAs), lipids, proteins (oncoproteins, tetraspanins, Rab GTPases, HSPs, HLA-Gs, and lectins), surface intercellular adhesion molecules (ICAM and integrins A and B), GFs (TGF-β, TNF-α, FGF2, and VEGF), selectins, cytokines (IL-6 and IL-8), and metalloproteases (Dilsiz, 2020; Jurj et al., 2020). The contents of EVs found in body fluids are closely related to the status of the producing cells, of which they can be biomarkers (Vader et al., 2014) and be recognized by or transferred to other cells in a selective manner, thus influencing the phenotype and functions of the recipient cells. In cancer patients, the quantity of circulating EVs seems to be higher than that in healthy subjects and has been found to correlate with poor prognosis (Kim et al., 2003; Vader et al., 2014). Depending on the tumor type and location, EVs can be isolated from plasma, serum, urine, body fluids, and even saliva (Vader et al., 2014). Tumor cells utilize EVs for dictating a defined tumor functional phenotype to surrounding cells (Naito et al., 2017; Jurj et al., 2020). Recent data show that tumor EVs contain molecules for intercellular communications (da Silva Nardi et al., 2016; Rebmann et al., 2016) that act on and impair recipient immune cells, favoring tumor initiation, growth, angiogenesis, immune surveillance, evasion, EMT, invasion, metastasis, and chemoresistance (Sheu and Shih, 2010; Kosaka, 2016). EVs (exosomes in particular) perform distinct roles during each of the sequential steps in the pre-metastatic niche evolution, namely, vascular leakiness, stromal cell education at organ-tropic sites, bone marrow-derived cell education and recruitment (Hoshino et al., 2015). Exosomes, with their contents, play roles in the sequential steps of the whole tumor process, from the primary tumor site modulation (such as the induction of angiogenesis, EMT, and immune suppression) to organ-specific metastasis homing, involving integrins, miRNAs, GFs, and growth factor receptors (GF-Rs) (Li et al., 2019). Exosomes interact with pre-metastatic niches, inducing angiogenesis, immune modulation, and reprogramming (Grange et al., 2011). Such an induced reprogramming activity by EVs could also be responsible for the conversion of non-CSCs into CSCs upon radio-chemotherapeutic treatments, which, in destroying CSCs, likely spread the EVs of these both in the tumor microenvironment and systemically (Chen et al., 2017; Ozawa et al., 2018; Keklikoglou et al., 2019; Lu et al., 2020). Moreover, exosomes help metastatic circulating cells in escaping immune surveillance and surviving in the blood circulation (Li et al., 2019). EVs from highly metastatic tumor cells have been shown to carry significantly different cargoes than do EVs from poorly metastatic cells (Rana et al., 2013; Vader et al., 2014). Exosomes reflect the status of donor cells, such as the hypoxic status of glioma cells, where they mediate the hypoxia-dependent activation of vascular cells during tumor development (Kucharzewska et al., 2013; Vader et al., 2014; Han et al., 2019).

## Embryonic Inductions and Tumor Inductions

Tumor progression necessarily requires the control and the overexploitation of the host surrounding microenvironment. To this end, tumor cells can dictate tumor functional phenotypes to surrounding cells through molecules for intercellular communications, such as HLA-G and exosomal miRNAs, as embryonic cells also do (da Silva Nardi et al., 2016; Rebmann et al., 2016; Dilsiz, 2020; Pillay et al., 2020). These molecules could induce such phenotypes by activating or silencing defined genic systems in the recipient cells.

### Embryonic Inductions and Histo-Organogenesis

The above inductive process could occur through EV contents (Sheu and Shih, 2010; da Silva Nardi et al., 2016; Rebmann et al., 2016; Naito et al., 2017; Jurj et al., 2020; Pillay et al., 2020) and be similar to the embryonic inductions occurring between mesenchymal and epithelial cells from early gastrula to organogenesis, during which the embryo structures progressively develop (Balinsky, 1970). It is known that embryonic inductions require a direct contact between mesenchymal and epithelial cells (Balinsky, 1970), as described in the SCID mouse model, between meta-nephric mesenchymal (MM) cells and ureteric bud (UB) epithelial progenitor cells in a three-dimensional co-culture, allowing for a direct cell–cell contact (Ratajczak et al., 2006; Valadi et al., 2007; Velagapudi et al., 2012). Here, a well-orchestrated series of reciprocal inductive events leads to the progressive formation of different structures of an early simple nephrogenesis (Velagapudi et al., 2012). Factors known to induce MM cells are present in UB cell-conditioned media and include several GFs, such as epidermal growth factor (EGF), TGF-α, basic fibroblast growth factor (bFGF), bone morphogenetic protein 7 (BMP7), hepatocyte growth factor (HGF), that could be indicated as “epithelial inductors.” Whereas, factors able to induce UB cells are present in MM cell-conditioned media and include the glial cell line-derived neurotrophic factor (GDNF), HGF, and extracellular matrix (ECM) proteins (collagen, fibronectin, and laminin), which could be indicated as “mesenchymal inductors” (Balinsky, 1970; Ratajczak et al., 2006; Velagapudi et al., 2012), with HGF as both an epithelial and mesenchymal inductor. These reciprocal inductions could trigger, in both epithelial and mesenchymal cells, a progressive consequent activation and/or silencing of defined genes, thus realizing a specific “inductive gene chain” (IGC) that finally confers to each cell of the generated progeny a distinct phenotype, resulting in a defined histological and physiological cell hierarchy in the histo-organogenesis process. The terminal IGC of each cell type would then be preserved by a defined genic homeostasis through genetic, epigenetic, and microenvironment signals.

### Tumor Inductions and Metastasis

Now, I hypothesize that such a type of intercellular induction could also occur in the cancer progression between mesenchymal CSCs (CSC_3_s) and surrounding cells, both tumor cells (CPCs and CDCs) and normal host cells (fibroblasts, macrophages, epithelial, and endothelial cells), through the EV contents of CSCs and CPCs, dictating new tumor-associated phenotypes. The induced cells would reciprocally supply their epithelial inductors to mesenchymal CSCs for generating oligopotent CPCs that, actively proliferating, would generate abundant different CDCs, thus determining a defined cell hierarchy and the histopathological features of a tumor. This process of consequential reciprocal inductions would occur through defined “on/off” switches, according to the genic program (IGC) of the origin cancer cell (CSC_0_). The level of realization of a defined IGC within the induced cells of a tumor would be responsible for their differentiation degree and, thus, for the different malignancy levels. I think that a crucial mechanism for the inductions in tumor progression is based on the cargoes of EVs (exosomes in particular) (Sheu and Shih, 2010; da Silva Nardi et al., 2016; Rebmann et al., 2016; Naito et al., 2017; Jurj et al., 2020; Pillay et al., 2020). In the recipient cancer and normal cells, exosomes deliver their contents modulating cell signaling pathways. Indeed, their repertoire of different components seems to constitute just a molecular machinery suitable for realizing cell induction processes for the growth and development of metastases, as equivalent to rudiments of organ portions (Velagapudi et al., 2012; Jurj et al., 2020). Cancer cells can produce about 10 times more exosomes than do normal cells. Released in the surrounding tumor microenvironment, exosomes have important roles in tumor initiation, progression, immunosuppression, neovascularization, metastasis, and drug resistance. Tumor cell-released exosomes can be taken up by the surrounding cells and travel through biological fluids, such as the blood, urine, and saliva (Dilsiz, 2020). Thus, cancer-associated EVs might exert systemic effects through the transfer of their cargoes, resulting in the reprogramming of recipient cells (stromal cells, immune cells, and bone marrow-derived cells) in the surrounding tumor microenvironment (Han et al., 2019). I believe that all this would reflect the induction phenomena in embryo development from the gastrula to the organogenesis phase (Balinsky, 1970), where direct cell-to-cell interactions between mesenchymal and epithelial cells occur and are indispensable for the pleiotropic development of defined biological structures.

## Properties and Roles of EV Contents in Cancer

### Integrins

Adhesion and ECM molecules, such as integrins, tenascin, and periostin, were shown to promote metastases of spreading cancer cells (Weaver et al., 1997; Oskarsson et al., 2011; Fukuda et al., 2015; Hoshino et al., 2015). On tumor-derived exosomes, a specific repertoire of ITGs has been detected, which dictates their adhesion to specific cell types and ECM molecules in particular organs. ITGs expressed on the exosome surface could be specific for a defined tumor kind: for example, the exosomal ITGs a6b4 and a6b1 are associated with lung metastases, while ITG a1b5 is linked to liver metastases (Hoshino et al., 2015; Sung et al., 2015; Li et al., 2019). ITGs might determine organ-specific metastatic sites, both controlling directional cell movements through tissues in association with fibronectin (Sung et al., 2015) and preparing pre-metastatic niches through the ITG-mediated fusion of exosomes with organ-specific resident cells, as well as likely reactivating stemness genes (OCT4, SOX2, NANOG, and KFL4) *via* S100 factors (Lu et al., 2020). Thus, tumor exosome ITGs might determine organotropic metastasis through sequential mechanisms for the organ-specific homing process (Valadi et al., 2007; Hoshino et al., 2015). For example, exosomes expressing ITG avb5 specifically bind Kupffer cells in the liver, while exosomes expressing ITGs a6b4 and a6b1 bind lung resident fibroblasts and epithelial cells (Hoshino et al., 2015). This specific organotropism and the related uptake of defined exosome ITGs promote, in the target cells, the upregulation of different pro-inflammatory and pro-migratory S100 genes in lung fibroblasts and Kupffer cells, but not in lung epithelial cells. Since S100A4 regulates lung metastasis and is controlled by ITG a6b4 (Hoshino et al., 2015), it has been suggested that this ITG activates the S100A4–Src axis in lung fibroblasts during the pre-metastatic niche formation (Hoshino et al., 2015). Moreover, since S100A10 facilitates OCT4-mediated breast cancer stemness (Lu et al., 2020), exosome ITGs could not only promote specific organotropic adhesion but also trigger the signaling pathways for stemness and reprogramming, as well as for inflammatory responses in target organs (Hoshino et al., 2015). Notably, exosomes secreted by a certain tumor are sufficient to redirect the organotropism of metastases of a different tumor type, normally unable to metastasize a particular organ (Hoshino et al., 2015). The exosomal ITG expression profiles of plasma exosomes isolated from cancer patients could be used for predicting sites of future metastasis and for developing diagnostic tests and therapeutic tools (Hoshino et al., 2015). After the ITG-mediated organotropic incorporation of tumor exosomes in recipient cells, ITGs have been shown to upregulate the expressions of several S100 genes related to cell migration and pro-inflammation (Hoshino et al., 2015). Now, since many different ITGs and S100 genes exist, I think that they, together with other factors, such as exosomal miRNAs, lncRNAs, and mRNAs, could constitute or be part of a cell-specific inductive machinery finally activating the IGC of a recipient cell at a defined genic level (Hoshino et al., 2015; Lu et al., 2020) related to the IGC of the tumor donor cell, thus determining the differentiation degree of the recipient cell.

### miRNAs

MicroRNAs represent the major class of small (20–22 nt), single-strand, non-coding RNA molecules. They are known to be fundamental regulators of gene expression in cancer cells, mainly as negative regulators of mRNA translation by binding to its complementary sequences (about 6–8 nt) into the 5′ or the 3′ region, thus leading to the degradation of specific target mRNAs or to the inhibition of their translation at a posttranscriptional level (Dilsiz, 2020). It is believed that miRNAs control about 60% of all the protein-coding genes in humans through miRNA–mRNA regulatory relationships, where many different miRNAs are often required to target a single mRNA molecule by a mechanism recognizing complementary sequences, with subsequent mRNA degradation or translation repression (Dilsiz, 2020). miRNAs are transferred to target recipient cells by exosomes, which protect them from degradation until their entry into target cells. Since cancer cells produce a high variety of exo-miRNAS that promote tumor proliferation, angiogenesis, and migration, these onco-miRNAs may be good biomarkers for many types of cancer, providing information about the identity of the type of cells releasing them and about their target cells (Dilsiz, 2020). Moreover, miRNAs in exosomes derived from leukemia cells (miRNA17–22 cluster) are involved in the migration and maturation of endothelial cells for cancer angiogenesis (Umezu et al., 2013; Jurj et al., 2020). Exo-miRNAs and other RNAs are also responsible for the activation or suppression of the innate and adaptive immune systems, such as miRNAs that act as TRL (Toll-like receptor) ligands in different types of cancer, stimulating their progression (Fabbri et al., 2012; Jurj et al., 2020). The exosomes of glioma stem cells with overexpression of miRNA-21 can be delivered to endothelial cells, thus stimulating neoangiogenesis, as exosomes from renal CSCs also do (Grange et al., 2011; Jurj et al., 2020). Exosome-specific miRNAs transferred from chemoresistant cancer cells in sensitive recipient cells can confer horizontal resistance through the modulation of drug-induced apoptosis, signaling pathways, and gene expression (Chen W. X. et al., 2014; Mao et al., 2016; Qin et al., 2017; Jurj et al., 2020). In breast cancer cells, exosomes contain different miRNAs that can modify the expression profiles of specific target genes, such as p27 by miRNA-24, as well as the chemoresistance by miRNA-5p (Mao et al., 2016; Jurj et al., 2020). Thus, miRNAs from tumor EVs might have several roles, such as silencing the genic program activities of recipient cells by repressing the translation of their mRNAs (Vader et al., 2014; Dilsiz, 2020) as well as inducing reprogramming at a genic level related to that of the donor cell (Kucharzewska et al., 2013), favoring angiogenesis for tumor progression and transferring horizontal chemoresistance.

### mRNAs

Messenger RNAs from tumor EVs would have the function of being translated into donor proteins (Ratajczak et al., 2006; Valadi et al., 2007; Vader et al., 2014) able to reactivate in recipient cells a genic program for inducing a new phenotype, with properties related to the status of the donor (Kucharzewska et al., 2013; Han et al., 2019). In effect, the mRNAs contained in microvesicles released by activated macrophages reflect specific phenotypes of the classically activated pro-inflammatory M1 or, alternatively, activated anti-inflammatory M2 macrophages (Garzetti et al., 2014; Jurj et al., 2020), thus indicating that the cell phenotype can be dictated by mRNAs transferred in recipient cells. Microvesicle mRNAs from colorectal cancer cells also promote the proliferation of endothelial cells (Hong et al., 2009).

### LIF, IL-6, and GFs

LIF, IL-6, GFs, and other factors could have the role of stimulating recipient cell growth and angiogenesis (Hood et al., 2009; Li et al., 2019), as well as of interacting with specific membrane receptors on target cells, thus activating defined endocellular pathways, such as the IL-6–gp130–JAK–STAT3 pathway. CSC-specific signaling proteins (β-catenin), specific surface receptors (CD133 and CD44), stem cell factors (OCT4), functional enzymes (ALDH), and transcription factors (TFs), which are activators of cell pathways all exported in exosomes, can mediate tumor stroma modulations by cancer cells and *vice versa* (Pavlides et al., 2009; Boelens et al., 2014; Richards et al., 2017; Jurj et al., 2020).

### HSP70s and HLA-Gs

HSP70s and membrane HLA-G1–4 on tumor cell would have a role of defense in the direct contact with immune [T lymphocytes (TLs) and natural killer (NK)] cells, neutralizing their cytotoxic activities or even inducing their apoptosis (Rouas-Freiss et al., 2003; Chalmin et al., 2010). HSP70s and soluble HLA-G5–7 released with tumor cell exosomes could favor and consent the invasion and colonization of host tissues in a systemic way, blocking all the components of the host immune system and enslaving other host cells for their ends (Rouas-Freiss et al., 2003; Sheu and Shih, 2010). The above series of events associated with EV contents clearly indicates that, in tumor progression, a real strategy exists for realizing an expansion–invasion–colonization process through cell inductions, which recalls those during embryo development. In this process, EVs and their contents could really be the crucial checkpoints in tumor progression since they can mediate and dictate the communications within the microenvironment both at the interface tumor/host tissues (Milane et al., 2015) and at a systemic level. In this strategy, each EV factor could carry on defined roles: defense from immune cells (HLA-Gs and HSP70s), detection of and adhesion to organ-specific pre-metastatic niches (ITGs), expression of oncoproteins in recipient cells (mRNAs), silencing and or activation of recipient cell genes (miRNAs and lncRNAs), and angiogenesis and growth (HLA-Gs, IL-6, LIF, and GFs).

## Current and Prospective Anticancer Strategies

During cancer development, different checkpoints could constitute potential therapeutic targets in the various phases of the process.

### Aspecific Targets

The following targets would be checkpoints theoretically associated with the development of different tumor types.

#### ID1, ID3, and CD44v

Inhibition of ID1 expression suppresses invasion and metastases in aggressive salivary and breast cancer (Fong et al., 2003; Murase et al., 2016). ID protein inhibition by a peptide aptamer induces cell cycle arrest and apoptosis in ovarian cancer cells (Mern et al., 2010a), and the inactivation of ID1 genes induces sensitivity of prostate cancer cells to chemotherapeutic drugs (Wong et al., 2008). Co-suppression of ID1 and ID3 significantly reduces proliferation, invasiveness, anchorage-independent growth, and angiogenesis and increases apoptosis in small-cell lung cancer (Chen D. et al., 2014); moreover, targeting ID1 and ID3 reduces the formation of peritoneal metastases by gastric cancer cells (Tsuchiya et al., 2005). Targeting ID1 and ID3 by a specific peptide aptamer induces E-box promoter activity, cell cycle arrest, and apoptosis in breast cancer cells (Mern et al., 2010b). Thus, targeting ID1 and ID3 could prevent growth, angiogenesis, and progression in primary avascular tumors and initial metastatic lesions. Knockdown of CD44 induces the differentiation of breast CSCs (Pham et al., 2011). CD44v isoforms are promising targets for the elimination of CSCs (Jin et al., 2006; Orian-Rousseau and Ponta, 2015; Yan et al., 2015). The inhibition of CD44v3 and CD44v6 by the A5G27 peptide copolymer blocks tumor invasion and metastatic colonization (Zaiden et al., 2017). Thus, targeting CD44v could exert synergic effects with ID1/ID3 targeting to block the stemness and migration of CSCs.

#### LIF/LIFr, IL-6, HLA-Gs, and HSP70s

LIFr knockdown inhibits the migration of melanoma cells in wound-healing tests (Guo et al., 2015). Neutralizing antibodies (Abs) knock down the activity or expression of LIF and reduce *in vitro* the stem cell-like properties of murine slow-growing CSCs (American association for cancer research, 2012). The conformational anti-HLA-G monoclonal antibody (mAbs) 87G, as well as IL-2, IL-12, and IL-15, restores the NK activity drastically inhibited by HLA-Gs (Tilburgs et al., 2015). HSP70s could be an immune therapeutic target in a wide spectrum of tumor types, and cmHsp70.1 mAbs can significantly reduce the bulk of mHSP70 + CT26 mouse colon tumors (Stangl et al., 2011). Thus, targeting the above factors could prevent the implantation of initial avascular tumors, their vascularization, and subsequent progression.

### Specific Targets

These targets would be checkpoints theoretically associated with a defined tumor type or even to a single tumor.

#### EVs

Extracellular vesicles are recently becoming an emerging target in cancer therapy, and currently, several clinical trials using exosome-based cancer therapy are ongoing (Vader et al., 2014; Abak et al., 2018; Chulpanova et al., 2018). The origin and concentration of circulating microparticles differ according to the type and evolution of cancers (Kucharzewska et al., 2013; Mege et al., 2016; Jurj et al., 2020). As natural carriers for diverse bioactive cargoes, EVs are potential vehicles for the delivery of many forms of therapeutic substances, including mRNAs, miRNAs, lncRNAs, proteins, and drugs (doxorubicin, paclitaxel, curcumin, and acridine orange) (Pascucci et al., 2014; Srivastava et al., 2016; Wang J. et al., 2016; Bunggulawa et al., 2018; Han et al., 2019). Exosomes can be isolated from a patient’s fluids and, after suitable modification, transferred to the same patient for a targeted cancer therapy. Studies have reported a significant higher efficacy of drugs loaded into exosomes when compared with free drugs and can cross biological barriers, even the blood–brain barrier (Yang et al., 2015; Dilsiz, 2020). One exosome-based therapeutic strategy is the inhibition of onco-miRNAs by the delivery of antagonist tumor-suppressive complementary miRNAs, injected either systemically or locally into the tumor (Dilsiz, 2020). Systemically injected exosomes targeted to the EGF receptor (EGFR) deliver antitumor miRNAs to breast cancer cells (Ohno et al., 2013; Jurj et al., 2020). Exosomes can be used as nanoparticles for suppressing the tumor growth and angiogenesis in gastric cancer by delivering HGF siRNAs (Zhang et al., 2018). Another original therapeutic strategy seems to be the removal of exosomes from circulation by extracorporeal hemofiltration or the prevention of the fusion and uptake of exosomes by target cancer cells (Marleau et al., 2012; Dilsiz, 2020; Jurj et al., 2020). On the other hand, exosomes can interfere with the activity of immunotherapeutic agents, such as therapeutic antibodies, which, a few hours after administration, result in approximately one-third to one-half bound to target cell exosomes (Battke et al., 2011). Human tumor-derived exosomes downmodulate NKG2D expression and inhibit the binding of Abs with tumor cells, reducing the antibody-dependent cellular cytotoxicity (ADCC) (Clayton et al., 2008; Ashiru et al., 2010; Battke et al., 2011). Similarly, tumor-derived exosomes participate in chemotherapeutic resistance by exporting certain drugs from cisplatin-resistant ovarian cancer cells (Safaei et al., 2005), thus impairing the endocellular activity of the drug.

#### ITGs

When uptaken in specific organs, tumor-derived exosomes prepare the pre-metastatic niche through distinct expression patterns of the ITGs associated with the metastases of defined organs. Targeting exosomal ITGs may effectively block organ-specific metastasis; targeting ITG a6b4, the exosome uptake and metastases in lungs decrease, as well as targeting ITG avb5 in the liver. Exosomal ITGs and exosome-inducible S100 molecules can constitute targets for an anti-metastatic combination therapy (Hoshino et al., 2015). ITG a6b4 controls the expression of genes associated with cell motility, invasion, and metastasis, including *S100A4*/*metastasin* (Grum-Schwensen et al., 2005; Chen et al., 2009; Kim et al., 2009; Lukanidin and Sleeman, 2012; Hoshino et al., 2015). In mice lacking the *S100A4* gene, tumor development and metastasis formation are suppressed (Grum-Schwensen et al., 2005; Lukanidin and Sleeman, 2012; Hoshino et al., 2015). Silencing of *S100A10* blocks the chemotherapy-induced enrichment of breast CSCs, impairs tumor initiation, and delays recurrence (Pillay et al., 2020). In three-dimensional culture and *in vivo*, reversion of the malignant phenotype of human breast cancer cells occurs by ITG blocking antibodies (Weaver et al., 1997; Hoshino et al., 2015).

#### miRNAs

Besides potential diagnostic and prognostic biomarkers in cancer monitoring, exo-miRNAs can be used in therapeutic strategies, such as the inhibition of onco-miRNA expression by the delivery of antagonist tumor-suppressor miRNAs: oligonucleotides complementary to the sequences of the targeted onco-miRNAs, loaded into exosomes, can be delivered both systemically and by local injections in the tumor bulk (Dilsiz, 2020). Tumor-suppressor miRNAs, loaded in exosomes and delivered, can inhibit pro-angiogenic mRNAs or knockdown specific genes for inhibiting tumor growth (Dilsiz, 2020); the exosome-formed synthetic miRNA-143 transferred to osteosarcoma cells inhibits their migration (Shimbo et al., 2014; Jurj et al., 2020). Notably, consistent with the para-embryonic nature of cancer, miRNAs have been shown in pregnancy, which may provide insights into a possible cure for cancer (Pillay et al., 2020). Cancer cell exosomes depend on cell surface heparan sulfate proteoglycans for their internalization and functional activity (Christianson et al., 2013; Vader et al., 2014), and heparin blocks EV transfer between donor and recipient cells (Atai et al., 2013; Vader et al., 2014).

Overall, ID1, ID3, CD44, LIF/LIFr, HLA-G, and HSP70 could be crucial aspecific targets mainly in primary tumor development, whereas exosome mRNAs, miRNAs, lncRNAs, ITGs, and related S100 factors might be important specific targets for blocking tumor progression and recurrence as well as for inhibiting organotropic metastasis locations.

## Discussion and Proposals: “Tumor Checkpoint Profiles” for “Shielded” Cancer Treatments

### Final Considerations

One first consideration about the aforesaid targets is that the non-specific checkpoints (ID1, ID3, CD44v, LIF/LIFr, HSP70, and HLA-G) would be theoretically associated with the development of different tumor types, while the specific checkpoints (miRNAs, lncRNAs, mRNAs, ITGs, and S100s) could be associated with a defined tumor type or, perhaps, to a single tumor. Thus, since these specific factors are circulating in biological fluids (Dilsiz, 2020) and reflect the status of tumor donor cells (Kucharzewska et al., 2013; Han et al., 2019), it would be important to detect and identify the different EV factors from tumors of the same kind, with similar differentiation degree and molecular features, for knowing whether common crucial characterizing checkpoints exist and act in the induction and maintenance of a specific phenotype in the CSCs and non-CSCs of similar tumors. To this end, it might be useful to collect, in a systematic manner (Table 1), all the data about each tumor in a sort of “cancer checkpoint hub” (Mege et al., 2016) in order to create a “molecular checkpoint profile” of each tumor type. This might allow characterizing the different tumor types, knowing their evolution, and detecting eventual common specific and aspecific targets suitable for aimed tumor treatments. In such a direction, it could be relevant to explore, for example, the potential properties of the ITG a2b1 as a potential marker and driver of all cancer metastasis types, protection from which could avoid all metastatic events (Hoshino et al., 2015).

**TABLE 1:** A very simplified model for the tabulation of tumor molecular checkpoints.

TUMOR TYPE	ASPECIFIC CHECKPOINTS					SPECIFIC CHECKPOINTS					OTHER CHECKPOINTS
	CD44v	ID1/3	HSP7O	HLA-G	LIF/LIFr	miRNAs	mRNAs	IncRNAs	lTGs	S1 00s	
BREAST TUMORS											
(a) ER/PR											
(b) HER—2											
(c) TN											
LUNG TUMORS											
.…….											
.…….											
………											
OTHER TUMORS											
……….											
……….											
……….											

*For each tumor type, the presence (+), absence (−), or the specific typology (i.e., CD44v3, CD44v6, etc.) might be indicated for each checkpoint. Eventual other checkpoints could also be inserted in the last column.*

Targeting the tumor cells immunologically with mAbs binding the complement (ADCC), or with other cytolytic immune mechanisms (NK cells and cytotoxic TLs), could result unfavorably (Wieckowski et al., 2009; Chen X. et al., 2018; Lin and Yan, 2019) since EVs released from killed tumor cells would spread in the microenvironment and in circulation, transferring their pathogenetic components to other cells, which might be induced to convert from non-CSCs into CSCs (Chen et al., 2017; Lu et al., 2020), enslaved to tumor ends (Sheu and Shih, 2010; Vader et al., 2014; Crange et al., 2015; Chen X. et al., 2018), or even killed by apoptosis (Lindman et al., 2006; Wieckowski et al., 2009).

Chemo- or radiotherapeutic treatments also could have the aforesaid unfavorable effects (Yan et al., 2005; Chen et al., 2017; Lu et al., 2020) since they might spread the cellular contents of the killed tumor cells, in particular EVs, which would enter other cancer and non-cancer cells, leading to the recurrence of the disease in time, as, in general, really occurs with these therapeutic treatments. In such a direction, it would be interesting to explore the blocking effect of heparin on the EV transfer between donor and recipient cells (Atai et al., 2013; Christianson et al., 2013) as a potential anti-EV molecular shield in cytotoxic cancer treatments.

Removal of immunosuppressive exosomes from the patient circulation by extracorporeal hemofiltration has been suggested and carried out as a therapeutic adjuvant in cancer treatment (Marleau et al., 2012). This idea appears to be good since such a technique could eliminate a likely crucial mechanism of tumor relapse and metastasis, in accordance with the above considerations.

The tumor bulk hierarchic structure, for which I suggested a modular organization of different cancer cell populations (Figures 1, 3) (Manzo, 2020), also might be a crucial problem to be considered in cytotoxic therapeutic strategies.

### “Shielded” Cytolytic Cancer Treatments

The emerging data about the induction properties of EVs and their contents in tumor progression and metastasis now allow a better view of the whole cancer process, where exosome contents appear to be crucial specific checkpoints in tumor immune evasion, recurrence, and metastasis. In this scenario, it seems very important to consider that any destruction of CSCs, by surgery or radio-, chemo-, or immunotherapy, could inevitably lead to disease recurrence and metastasis because of the induction of stemness by EV contents (Yan et al., 2005; Chen et al., 2017; Lu et al., 2020). Therefore, it would be indispensable to carry out cytotoxic antitumor treatments under protection from this activity, on the basis of the molecular checkpoint profile of a defined tumor type. To this end, the following general multistep therapeutic strategy could be proposed for back-dismantling the tumor hierarchic histological structure, preventing recurrence and metastasis:

Step 1: Depletion of non-proliferative differentiated tumor cells (CDCs) by conventional chemotherapeutic agents (Wang T. et al., 2016; Roy et al., 2018) and/or radiotherapy, killing different bulk cancer cells. Since non-specific chemotherapies or radiotherapy, besides CDCs, also kill random CSCs, releasing exosomal factors with stemness induction properties (Chen et al., 2017), these cytolytic treatments should be effectuated under protection from whole exosomes (for example by hemofiltration or heparin) (Atai et al., 2013; Christianson et al., 2013) and free exosomal factors (for example by anti-HLA-G/HSP70/ITG/S100 mAbs or by miRNAs targeting onco-RNAs) (Weaver et al., 1997; Stangl et al., 2011; Hoshino et al., 2015; Tilburgs et al., 2015; Dilsiz, 2020) to avoid immune system impairment, non-CSC/CSC transition (recurrence), and pre-metastatic niche induction (metastasis).
Step 2: Depletion of actively proliferating tumor cells (CSC_2_s and CPCs) by cell cycle-independent apoptotic drugs, like alkylating agents (Wang T. et al., 2016; Roy et al., 2018), yet under a protected approach, as in step 1.
Step 3: Elimination of slow- and non-proliferating CSCs (CSC_1_s and CSC_3_s), always under a shielded approach, like in step 1, by anti-CSC chemotherapeutic agents (Wang T. et al., 2016; Roy et al., 2018), mi-Abs or aptamers specifically targeting ID1, ID3, CD44, and LIF/LIFr (Heap et al., 2005; Pecak et al., 2020), or also in a natural way by NK cells that preferentially target tumor cells with a CSC phenotype (Ames et al., 2015), now easily accessible in the tumor site, after the depletion of CSC_2_s, CPCs, and CDCs.

## Data Availability Statement

The original contributions presented in the study are included in the article/supplementary material, further inquiries can be directed to the corresponding author/s.

## Author Contributions

The author confirms being the sole contributor of this work and has approved it for publication.

## Conflict of Interest

The author declares that the research was conducted in the absence of any commercial or financial relationships that could be construed as a potential conflict of interest.
